# A Simplified, Light Emitting Diode (LED) Based, Modular System to be Used for the Rapid Evaluation of Fruit and Vegetable Quality: Development and Validation on Dye Solutions

**DOI:** 10.3390/s150922705

**Published:** 2015-09-08

**Authors:** Raffaele Civelli, Valentina Giovenzana, Roberto Beghi, Ezio Naldi, Riccardo Guidetti, Roberto Oberti

**Affiliations:** Department of Agricultural and Environmental Sciences, Università degli Studi di Milano, Via Celoria 2, Milano 20133, Italy; E-Mails: raffaele.civelli@unimi.it (R.C.); valentina.giovenzana@unimi.it (V.G.); ezio.naldi@unimi.it (E.N.); riccardo.guidetti@unimi.it (R.G.); roberto.oberti@unimi.it (R.O.)

**Keywords:** portable optical device, non-destructive analysis, reflectance, ripening, fruit and vegetable

## Abstract

NIR spectroscopy has proven to be one of the most efficient and ready to transfer tools to monitor product’s quality. Portable VIS/NIR instruments are particularly versatile and suitable for field use to monitor the ripening process or quality parameters. The aim of this work is to develop and evaluate a new simplified optoelectronic system for potential measurements on fruit and vegetables directly in the field. The development, characterization and validation of an operative prototype is discussed. LED technology was chosen for the design, and spectral acquisition at four specific wavelengths (630, 690, 750 and 850 nm) was proposed. Nevertheless, attention was given to the modularity and versatility of the system. Indeed, the possibility to change the light sources module with other wavelengths allows one to adapt the use of the same device for different foreseeable applications and objectives, e.g., ripeness evaluation, detection of particular diseases and disorders, chemical and physical property prediction, shelf life analysis, as well as for different natures of products (berry, leaf or liquid). Validation tests on blue dye water solutions have shown the capability of the system of discriminating low levels of reflectance, with a repeatability characterized by a standard deviation proportional to the measured intensity and in general limited to 2%–4%.

## 1. Introduction

The study of non-destructive methods and the design of new devices for monitoring a large number of samples in a short time and allowing a more comprehensive overview of ripening is an ongoing research subject [1,2,3].

To this aim, currently visible near-infrared (VIS/NIR) and near-infrared spectroscopy are techniques widely applied in the food sector [4]. A review of the literature reveals that NIR techniques (VIS/NIR and NIR) have been applied to a wide range of agri-food applications. The feasibility of NIR spectroscopy to measure quality attributes of fruit and vegetables has been shown for many products [2]. Data complexity arising from NIRs requires specific statistical analyses and qualified operators. Furthermore, nowadays, the available devices are quite expensive and therefore not suitable for small-scale producers.

During fruit ripening, biochemical changes occur not just at the skin level, but also in the pulp. NIRs analyses allow one to reach the inner layers of the sample when appropriate wavebands are used. For these reasons, NIR spectroscopy resulted in being a suitable technique to evaluate ripeness in the orchard and post-harvest quality characteristics of fruits.

To this aim, three main types of NIR devices can be identified: (i) laboratory instruments for applications in research centers or in industry laboratories; (ii) sorting and grading devices designed specifically for the fruit and vegetable industries, e.g., in warehouses; (iii) portable devices for use directly in the field. Table 1 shows the main differences between the three types of NIR devices.

**Table 1:** Characteristics of the three main categories of NIR devices.

	Application Area	Flexibility of Use	Applicability	Measurement Accuracy and Reproducibility	Cost
**Laboratory devices**	Research/Industry	Adaptable to different matrices	Fixed system	Optimal	Average/high
**Sorting and grading**	Industry	Specific categories of products	Fixed system	Fair	Average/high
**Portable devices**	Also in field	Dedicated for individual products	Portable/handheld	Fair	Average

Nowadays, a wide selection of spectroscopy devices at different complexity levels is available, and there are about 60 NIR spectrometer manufacturers around the globe [5].

For every device category, calibration models to be used in practice need to be based on large datasets, encompassing several orchards, cultivars, growing conditions, seasons and device operating conditions (e.g., temperature, environmental lighting). All of these factors have to be incorporated within appropriate preprocessing methods or to be compensated [2,3], aiming at optimizing prediction robustness. Sample presentation to the instrument is also a crucial step in NIR analyses. Specialized sampling probes, liquid cells and accessories have been manufactured to meet measurement demands according to how and where they will be performed (e.g., in a laboratory, at a production line or in the field) [3,6].

The availability of handheld spectrophotometers has opened up the possibility to use them in the orchard for monitoring fruit maturity. Scientific literature reports some studies on applications of portable NIRs to this aim. In most cases, limited prediction accuracy was obtained. For example, by using a portable VIS/NIR instrumentation, Antonucci *et al.* [7] predicted the titratable acidity on two cultivars of mandarin getting $R^{2}$ a range of 0.66–0.77, while for the soluble solids content, a $R^{2}$ range of 0.71–0.72 on apricot. Camps and Christen [8] estimated the soluble solids content, the total acidity and the firmness achieving $R^{2}$ in cross-validation in ranges of 0.77–0.92 0.53–0.94 and 0.72–0.85, respectively. Beghi *et al.* [9] on golden delicious apples, determined $R^{2}$ the soluble solid content, chlorophyll, titratable acidity, flesh firmness,total phenols, carotenoids and ascorbic acid, equal to 0.72, 0.86, 0.52, 0.44, 0.09, 0.77 and 0.50, respectively.

This might be due to factors affecting model robustness, such as temperature fluctuations, uncontrolled lighting conditions, the limited wavelength range or the fact that the devices were still at a prototype stage. The development of portable devices suitable for field use is more complex than laboratory applications, due to the uncontrolled environmental conditions, such as ambient light, fluctuating temperatures, power source, illumination, but also the need of instrumentation with a lower technological complexity. All of these phenomena should be addressed by appropriate data processing [2].

Portable VIS/NIR instruments were tested in controlled laboratory conditions by Antonucci *et al.* [7] for the evaluation of mandarin maturity status and by Camps and Christen [8] for assessing apricot quality. Under more difficult uncontrolled field conditions, instead, a portable VIS/NIR device was tested in order to estimate apples’ nutraceutical properties [9], to evaluate grape quality parameters [10], to assess the ripeness of red-pigmented fruits [11] and to predict blueberry ripeness [12].

Research and innovations have enabled NIRs devices to further decrease their physical size while increasing complexity and the amount/size of collected data. Therefore, new NIRs instrumentation tends to be more compact and portable [3,5] opening new possibilities for field use.

Few portable commercial spectrophotometers, for example the Fruit Tester 20 (FANTEC, Kosai-city, Japan) [13], the Jaz Modular Optical Sensing Suite (Ocean Optics Inc., Dunedin, FL, USA), and the QS_300 (Unitec SpA, Lugo, Italy), have been proposed for different food applications.

For each device category (Table 1), the development of calibration models and the extraction of useful information contained in spectral data rely on multivariate analysis. Chemometrics, in fact, is a crucial part of NIR spectroscopy applications. Indeed, these measurement techniques must always be complemented with chemometric analysis to enable the extraction of useful information present in the spectra, by separating it both from useless information or from spectral noise [2,14,15,16].

Furthermore, the use of portable NIR devices requires skilled operators, able to process complex data in order to extract useful information and to build ripening prediction models [4,16]. Therefore, in order to support farmers or small-scale producers, simplified, easy to use and low-cost devices for real-time measurements in field are desirable.

To reach this goal, considerable efforts have been recently directed towards developing and evaluating different procedures for an objective identification of a few spectral variables containing most of the useful information and to reject redundant or useless variables [17]. In view of simplified optical systems, different approaches for variables selection were applied in previous studies conducted by the authors on fresh-cut *Valerianella* [18], on wine grapes [19] and on blueberries [20].

Only a few examples of simplified optical systems have been reported in the literature. For example, the University of Bologna [21] patented a new simplified NIR device in a two-fold version (DA-Meter for apples, and Kiwi-Meter for kiwi fruits, where DA refers to a maturity index introduced by the authors based on the difference in absorbance between specific wavelengths). These devices evaluate the fruit maturity stage through indices based on absorbance differences among specific wavelengths.

The aim of this work was to develop a simplified four-wavelength optical system, based on LED technology, potentially to be used for the rapid monitoring of fruit and vegetable parameters directly in field (e.g., ripeness evaluation, disease and disorder detection, chemical and physical property prediction, freshness level or shelf life analysis). In order to assess the performance of the prototype, the characteristic curve and the operating point were investigated, and tests on standard solutions were performed.

## 2. Experimental Section

The design of the measurement system pursues different objectives: low cost, low complexity (thanks to the use of a microcontroller), modularity, tuning possibilities and versatility, *i.e.*, the capability to adapt to the different needs required by a specific application. The system is based on the acquisition of the spectral reflectance at wavelengths of interest. Light emitting diode (LED) technology was chosen as the light source [3], so as to achieve the capability to individually adjust the light emission intensity for each measurement channel.

### 2.1. System Hardware

**Figure 1:**
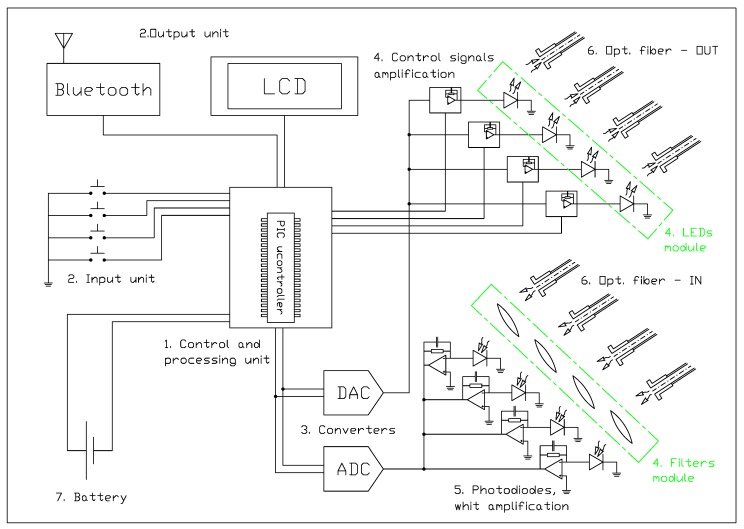
Block diagram of the main components of the prototype.

Figure 1 shows a diagram of the instrument with the component units in relationship to their functionalities. In particular, as shown by the figure, the system is composed of:

1. the control and processing unit;
2. the interface unit;
3. the analog-to-digital and the digital-to-analog converters;
4. the LEDs and optical filter modules;
5. the photodiodes;
6. the eight-arm optical fiber;
7. the power supply system.

#### 2.1.1. The Control and Processing Unit

This is the main board of the device, equipped with a PIC^TM^ microcontroller (Programmable Integrated Controller, PIC18F series, Microchip Technology Inc., Chandler, AZ, USA), (1 in Figure 1). When the instrument is turned on, the microcontroller automatically runs a preloaded firmware for the initialization, and manages the inputs entered by the user. Based on these, it checks the state of the system and sends signals to the other units in order to operate. The main program was written using a development tool based on a C language compiler and then stored on the memory of the microcontroller.

#### 2.1.2. The Interface Unit

The user can control the system through a four-button keyboard connected to the main board (2 in Figure 1), which represents the input interface. By using the keyboard, the operator can control different tasks, e.g., he can start the calibration procedure, make a new measurement or delete the last one.

The output interface is an alphanumeric four-line LCD display (2 in Figure 1). The acquired data and other messages are displayed in real time, giving the user instantaneous feedback to check if the instrument is working correctly. At the same time, the data are sent to a palmar tablet or a notebook PC through a wireless Bluetooth card. In this way, the work session can be followed on a PC monitor, and data can be saved for any post-processing.

#### 2.1.3. The Analog-to-Digital and the Digital-to-Analog Converters

The communication between a digital component, such as the microcontroller in the control unit, and the actuators (LEDs) or the transducers (photodiodes) requires the conversion of the voltage/current signals from digital to analog form and *vice versa*.

This task is accomplished by the 8-bit digital-to-analog converter (DAC; 3 in Figure 1), for the modulation of the LEDs’ driving current and the 16-bit (1 bit for the number sign, 15 bits for the encoding of the numerical value) analog-to-digital converter (ADC; 3 in Figure 1), which converts the analog inputs measured by the photodiodes in digital form. Both of these devices use the I^2^C^TM^ (Inter-Integrated Circuit) serial interface as the communication protocol.

#### 2.1.4. The LEDs and Optical Filters Modules

The instrument can be equipped with up to four independent light sources, whose luminous intensities can be individually adjusted and set. The requirement for accurately driving the luminous radiation within a wide range of values has been met using LEDs as light sources. In this way, according to the specific application, the system can emit a custom 4-wavelength spectrum, e.g., the analysis of red grape needs a high illumination intensity close to the anthocyanins absorption peak (around 520 nm), while it requires a low intensity near the third-overtone of the OH bond (around 740 nm).

The choice to limit the number of the measurement channels (channel = LED + filter + photodiode) to four has been established to reduce the complexity of the overall optical system, since this must be simple and handheld.

The current configuration of the device is based on the spectral bands selected in previous studies [19]. The LEDs actually used were chosen at the wavelengths that most closely matched the theoretical choice, according to the availability on the market. Therefore, the wavelengths used for the system are: 630 and 690 nm, near the chlorophyll absorption peak, 750 nm, near the third-overtone of OH bond stretching, and 850 nm, arbitrarily elected among the uninformative wavelengths for normalization purposes.

All four LEDs mounted in the prototype come from the same LED series (ELJ series, Roithner Lasertechnik GmbH, Vienna, Austria). These are high power LEDs designed for several optical applications, including use in measurement systems. The LED technology is particularly appropriate to be adopted in the present device. This is explained point by point by the following arguments, with reference to the technical data summarized in Table 2.

- The ELJ family is based on semiconductor materials (e.g., aluminum-gallium-indium-phosphide or gallium-aluminum-arsenide) that ensure highly accurate and well-defined wavelengths for the emitted radiations in the VIS/NIR range, with a narrow bandwidth. The peak wavelength tolerances do not exceed ±10 nm, with bandwidths ranging between 20 and 40 nm. Any possible shift of the emission wavelength due to the drive current changing is minimal (in the order of 1–3 nanometers). This effect is further compensated along every measuring channel by the introduction of a dedicated filter, which will be discussed later.
- The light intensity emitted by an LED is easily modulated by using the drive current with a fairly linear response (the study of the linearity of the overall response for the optical channel, *i.e.*, the characteristic curve, is among the objective of this work). This aspect, together with the reduce switching time, makes an LED particularly suitable to be controlled by the current signals coming from the microcontroller. Furthermore, the individual control of each LED makes it possible to generate a customizable 4-wavelength spectrum of emission, as stated above. Thanks to the use of LED technology, the channels can be independently managed, and the measure settings can be individually optimized.
- The light is emitted by an LED according to a narrow optical cone, which simplifies its collimation towards the optical fiber and reduces any possible crosstalk effects between the different channels. In particular, the LEDs of the ELJ collection are provided in a metal case with a plastic lens, which produces a viewing angle between 15° and 20° according to the model.
- The ELJ series is characterized by a good efficiency, which means high values for radiant power and intensity *versus* a relatively small dissipation power. Efficiency is useful with the intent of battery saving for applications in the field [2]. Typical values of the radiant intensities and radiant powers for a drive current at zero wavelength shift are reported in Table 2, and so the maximal values of the power dissipation.
- Finally, the compact sizes and the low weight of LEDs give an additional argument to choose this technology for the development of a handheld and portable system.

**Table 2:** Technical data for each measurement channel LED (Roithner Lasertechnik GmbH), filter (Edmund Optics Inc.) and photodiode (Roithner Lasertechnik GmbH).

Item		LED Model			
		ELJ-630-628	ELJ-690-629	ELJ-750-629	ELJ-850-629
Semiconductor material	-	AllnGaP	AlGaAs	AlGaAs	AlGaAs
Drive current at zero wavelength-shift $I_{0}$	mA	350	350	350	350
Max drive current $I_{M}$	mA	1000	700	1200	1200
Max power dissipation	mW	3000	3200	4000	4000
Peak wavelength ^*a*^	nm	630 ± 5	690 ± 10	755 ± 10	850 ± 10
FWHM^*b*^ Spectral bandwidth at 50 ^*a*^	nm	20	23	30	40
Viewing angle ^*a*^	deg	15	17	20	20
Radiant power ^*a*^	mW	95	55	80	100
Radiant intensity ^*a*^	mW/sr	830	380	650	2000
Switching time ^*a*^	ns	60	45	60	20
**Item**		**Filter Model**			
		**#65-651**	**#65-660**	**#67-775**	**#67-785**
Center wavelength	nm	632 ± 2	694 ± 2	750+3/−0	850+3/−0
FWHM ^*b*^	nm	10 ± 2	10 ± 2	10 ± 2	10 ± 2
Minimum transmission	%	≥45	≥50	≥50	≥50
Blocking wavelength range	nm	200–1200	200–1200	200–10000	200–10000
**Item**		**Photodiode Model**			
Spectral range ^*c*^	nm	400–1100			
Maximum spectral responsivity $S_{\max}$ ^*c*^	mV/nW	60			
Wavelength at $S_{\max}$ ^*c*^	nm	800			
Viewing angle ^*c*^	deg	±50			

${}^{a}$ Typical values at 20 °C and for $\mathrm{Drive}\;\mathrm{current}=I_{0}$, *i.e.*, drive current at zero wavelength shift; ${}^{b}$ full width-half maximum; ${}^{c}$ typical values at 25 °C and for supply voltage VS=±15 V.

Four optical filters (#65- and #67-series, by Edmund Optics Inc., Barrington, NJ, USA), one for each LED, are positioned at the end of the optical fiber input arms (see Section 2.1.6), just before the photodiodes. Each filter was chosen with a center wavelength as close as possible to the peak emission wavelength of the corresponding LED, according to the availability on the market (see Table 2). The #65- and#67-series have a narrow and well-defined spectral bandwidth of (10 ± 2) nm and ensure a transmission coefficient ≥45% , while filtering all other wavelengths in the visible and near-infrared range. The introduction of the filters cuts down the wide-range environmental noise during the acquisition, resulting in an improvement of the signal-to-noise ratio.

The LEDs and the relative filters represent the module unit of the instrument (4 in Figure 1), which can be changed according to the specific application (see Section 2.2).

#### 2.1.5. The Photodiodes

Each measurement channel is equipped with a silicon-based photodiode (IQ800L, Roithner Lasertechnik GmbH, Vienna, Austria) operating in the spectral range 400–1100 nm, with a peakresponsivity at 800 nm (see Table 2). Although the IQ800L is already provided with an integrated low noise JFET-amplifier (junction gate field-effect transistor amplifier), an external dedicated stage of electronic filtering and amplification was designed for every photodiode in order to further improve the signal-to-noise ratio, before the analog-to-digital conversion (5 in Figure 1).

#### 2.1.6. The Eight-Arm Optical Fiber

A customized optical fiber was specifically designed for the application in order to optimize the radiation transmission and the optical couplings, and an industry leader was commissioned for its realization.

**Figure 2:**
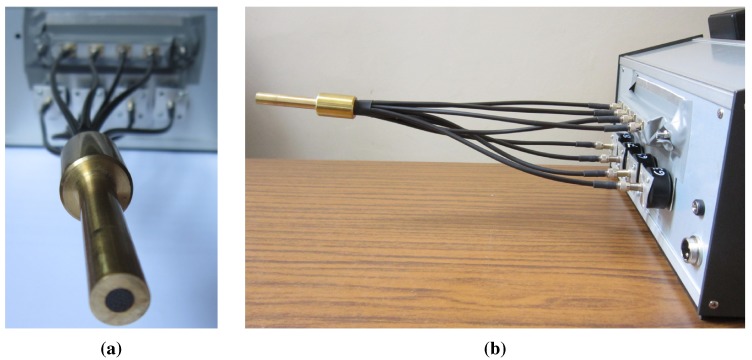
The customized quartz optical fiber: (**a**) front and (**b**) lateral view.

This consists of a bundle of 32 multimode step index fibers with a low OH fused silica core and glass cladding (Fort Fibre Ottiche S.r.l., Curno, Italy), with a diameter of 600 μm and a length of 30 cm arranged in 8 independent arms in protective plastic sheaths, of 4 fibers each (see the pictures in Figure 2). The arms are free at one end, each one with a standard SMA connector (subminiature version A coaxial connector), while at the other end, they all converge together in a single metallic probe where the fibers are mixed. This is the element that is put in contact with the sample during a measurement. Four arms (output arms OUT; 6 in Figure 1) are connected to the LEDs, receive the emitted radiation and transmit it towards the probe and then to the sample. Conversely, the other four arms (input arms IN; 6 in Figure 1) collect the radiation coming back from the sample and transfer it to the four photodiodes for the measurement.

### 2.2. Modularity

The prototype of the simplified optical device is designed with particular attention to versatility and modularity concepts. It is desirable to have the possibility to adapt the simplified optical device for different objectives, applications and different kinds of sample matrices, while keeping its main architecture.

This can be obtained by selecting the emission spectrum at specific wavebands by an appropriate choice of the LEDs. Envisaged applications for this kind of instrument would be, for example, the assessment of the main technological and phenolic parameters of grapes for wine production [10] or the early detection of disease infection (e.g., *Botrytis cinerea* or powdery mildew) on leaves [22,23] or berries.

### 2.3. How the System Works

Since the very beginning of the development of the device, the approach of sequential (multiplexed) measurements was preferred over the simultaneous one. This was mainly due to both optical and electronics reasons. From the optical point of view, the possibility to customize the emission spectrum by changing LEDs and filters (*i.e.*, modularity concept) makes potentially possible applications based on the investigation of very close wavebands, in particular to estimate the values of derivative spectra. The sequential design is therefore mandatory to mitigate the cross-talk effect due to the partial overlap of the emission/measuring spectral curves, which instead would occur with a simultaneous acquisition. Regarding the electronics aspects, the simultaneous activation of all channel LEDs was avoided for not incurring current load peaks in the system that could cause electronic inter-channels interferences, and, more in general, for power saving reasons in a portable, battery-powered device.

According to the acquisition procedure, when the user presses the dedicated button (2 in Figure 1), the microcontroller runs a memory pre-loaded routine, which controls the individual powering of the LEDs, according to a specific sequence. With reference to Figure 1, the acquisition routine can be schematically described as follows:

1. The microcontroller (1) sends the driving signal to power one of the LEDs.
2. As soon as the individual LED turns on, the emitted light is brought to the sample through the corresponding output arm of the optical fiber. The radiation interacts with the sample matrix, and the reflected back-scattering light is collected by the input arm (6).
3. At the same time, the microcontroller enables the detection on the corresponding photodiode (5) for an instantaneous acquisition. The output signal of the photodiode is than amplified and digitized by the ADC (3) and finally acquired by the control and processing unit of the microcontroller.
4. The measurement at one wavelength is obtained as an average of 5 acquisition cycles as repeated in sequence by the processor of the microcontroller. If the deviation among repetitions is higher than 3% of the average, the measurement is rejected and the sequence repeated.
5. The same steps (1)–(4) are sequentially repeated for all of the channels.
6. Once this procedure is completed for all of the LEDs, the data are ready to be shown on the display (2). In the same way, they are sent to the Bluetooth card (2) and then to the computer. The overall acquisition procedure takes less than 1500 ms, after which the device is ready for a new acquisition. A single acquisition is in practice almost instantaneous, considering that the device is intended for manual use in static applications.

### 2.4. Setting Parameters and Operating Characteristic Curves

The prototype is designed to perform a direct measurement of the reflected light intensity at specific wavelengths, expressed in an arbitrary unit coming from the electronics of the system (in a range between 0 and 32767).

The quality of the direct measurement is a prerequisite to obtain the correct estimation of the reflectance, *i.e.*, the ratio at a given wavelength between the intensity of the radiation reflected by the sample and the intensity of the illuminating radiation. The reflectance is the real significant variable, which allows comparisons between acquisitions made at different times and under different environmental conditions. The choice of the device setting parameters affecting the measurement output becomes therefore crucial.

The setting parameters should be chosen in order to establish a proper operating point along the characteristic curve of the system for each channel, both by ensuring the linearity of the response and by expressing an adequate sensitivity with respect to the specific application. In particular, measuring the same sample under the same conditions, the instrumental parameters, which can be set and that influence the detected light intensity on a certain channel, are two:

- The stimulation level of the LED drive current, Stimulus, which modulates the intensity of the incident light and, consequently, of the reflected light, as well;
- The amplification factor of the signal detected by the photodiode, Gain, which defines the numerical value actually read within the available measurement scale.

Following this, the response of the device as a function of the Stimulus and Gain parameters, *i.e.*, the characteristic curve of every channel of the system, was investigated, in view of the definition of a calibration procedure for setting an appropriate operating point, optimal for the application of the device.

A special test software has been implemented and loaded into the microcontroller to automatically explore the totality of the operating parameters space. With this configuration of the device, it was possible to evaluate the response for all of the admissible (Stimulus,Gain) couples (with integer values of Stimulus in the range 0<Stimulus<255 and Gain=1,2,4,8). The tests were repeated on the four available standard references (Spectralon^®^ Diffuse Reflectance Standards, Labsphere Inc., North Sutton, NH, USA), for reflectance coefficients R=0.99,0.50,0.25,0.02.

### 2.5. Identification of a Simple Calibration Procedure for Defining the Operating Set-Point

In order to simplify the use of the instrument, the full capability of the setting parameters (Stimulus,Gain) is not given to the user. These operating parameters have in fact to be properly set at the beginning of every working session, when the instrument is switched on. However, their identification, *i.e.*, the setting of an operating point along the characteristic curve for each channel, is application specific and in general not immediate.

For example, when measuring a dark-colored fruit or cultivar, like red grapes or blueberries, a high sensitivity through all of the visible range will be in general required. On the contrary, for a light-colored fruit, e.g., white grape cultivars, a high sensitivity in the red spectral range will be necessary (due to chlorophyll absorption in this range), while a low sensitivity at other visible wavelengths will be in general necessary; or again, when measuring green leaves, sensitivity needs to be optimized for different wavelengths: a partially low sensitivity will be required in the green range due to relatively high leaf reflectance; while a high sensitivity in the red band and low sensitivity in the near-infrared range will be necessary. Starting from the study of the characteristic curves, a simple calibration procedure, based on acquisitions made on standard reflectance references was defined (see Section 3.2) to properly set the operating point for each channel.

### 2.6. Validation Tests on Custom Samples

The device was then tested on three custom standard samples, consisting of distilled water solutions of blue food dye (Brilliant Blue R, CAS Number 6104-59-2, Sigma-Aldrich Co. LLC., St. Louis, MO, USA) at concentration of 0.5% (w/v), 1% (w/v) and 2% (w/v), respectively.

An acquisition setup for this kind of liquid sample was defined. According to this, a solution sample is inserted in a quartz 5 mm path cuvette (100-QS, Hellma GmbH & Co., Mullheim, Germany), which is then placed into a custom holder made of Teflon, *i.e.*, from a commercial-grade PTFE bar, externally covered with black cladding. A hole on the holder allows one to insert the optical fiber probe, guiding it to face the cuvette window, into a proper fixed position, which ensures repeatable measuring conditions. The cuvette containing the dye solution is therefore embedded into a diffuse reflecting material. As a result, the detected photons passed through the sample before and after being reflected in the direction of the detection fibers, without being absorbed by the sample. As the sample holder is fixed for all measurements, the dye concentration is the only variable property on the sample side.

Two kinds of tests were conducted.

First, the same explorative configuration presented in Section 2.4 was applied, and the characteristic curves of the system were built, by measuring the three standard solutions while changing the Stimulus parameter at the fixed Gain value, previously determined (Gain=1). These experiments aimed to assess the capability of the device of measuring differences in reflectance at a very low level of intensity (*i.e.*, at very high absorption). These conditions are expected, for example, when measuring dark fruit species, e.g., red grapes or blueberry, in the channels at 630 and 690 nm.

Secondly, the calibrated configuration of the instrument, ready for the operation, was validated by simply iterating five acquisitions on the standard solutions and evaluating the measurement repeatability. As described above in Section 2.5, each of the four channels followed an independent calibration with a dedicated reference.

## 3. Results and Discussion

### 3.1. Operating Characteristic Curves

**Figure 3:**
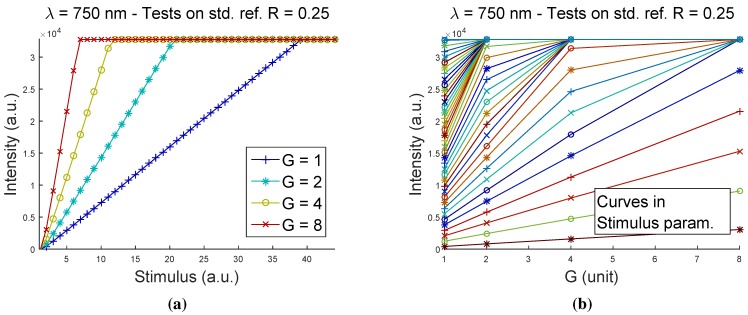
Acquisitions on channel λ=750 nm, using reference R=0.25: (**a**) output Intensity *vs.* Stimulus for the four curves in the Gain(G) parameter; (**b**) output Intensity *vs.* Gain(G) in the Stimulus parameter.

Each of the four channels has proven to have a similar characteristic curve. As an illustrative example, Figure 3 shows the output intensity obtained at the channel λ=750 nm when measuring a standard reference target with a reflectance R=0.25, respectively, while the Stimulus was changed at fixed Gain values (Figure 3a) and while the Gain was changed at fixed Stimulus values (Figure 3b).

A similar behavior was obtained for all four channels. In this regard, the measurement raw files coming from these tests are accessible as supplementary materials (refer to Appendix A, Appendix B, Appendix C and Appendix D).

Figure 3a represents the output intensity measured as a function of the Stimulus variable, for the four levels of Gain that the system allows to set. Only the significant portion of the graphs is displayed, *i.e.*, below Stimulus values causing saturation of the output signal. The characteristic curve of the system’s output intensity as a function of the Stimulus variable clearly exhibits a highly linear behavior, regardless of the Gain value set. As expected, higher values of Gain correspond to an increase in the slope of the linear curve.

The linearity of the response with the change of the Stimulus variable is then assured for every choice of the Gain parameter and, at least in theory, of the operating point position along the curves between zero and saturation.

Nevertheless, saturation is reached very quickly, and actually, just a small portion of the available range 0<Stimulus<255 is used. The highest slope curves (high Gain values), combined with highly reflective standard references could lead to the risk of reaching saturation even at the very first Stimulus values.

The phenomenon is pronounced in measurements on a standard reference with a higher reflectance, and it is in general confirmed by the other tests (refer to Appendix A, Appendix B, Appendix C and Appendix D). This is a common behavior in all four channels due to the optical high efficiency of the system.

In practice, Gain=1 is the only acceptable value for all four channels. It corresponds to the smallest slope curve, developing in the wider range of Stimulus values before saturation is reached.

Figure 3b leads to conclusions consistent with the previous ones. This plot shows the same acquisition (λ=750 nm and standard reference R=0.25) representing the output intensity measured as a function of the Gain variable, for the 256 curves parameterized in Stimulus. In this case, only the curves corresponding to the first values of the Stimulus parameter are distinguishable. All of the other curves collapse on the horizontal saturation line (Intensity=32767 a.u.) as the Stimulus parameter increases; they are therefore overlapping. The linearity of the response with the change of the Gain variable is very good, clearly for the curves not deformed by saturation effects (for a low value of Stimulus). A single acceptable value Gain=1 for all of the measurement channels is confirmed by this results, being the one providing the greatest number of significant Stimulus values.

Concerning the comparison among the different channels, the system shows the same kind of response for all four wavelengths (Appendix A, Appendix B, Appendix C and Appendix D). However, parameter values change in general depending on the specific channel. This is due to the LEDs and photodiode features that change depending on the operating wavelength, despite these components all coming from the same collections, as declared by the manufacturers. This aspect is taken into account by simply making an independent calibration on every channel using different standard references. Furthermore, this optimizes the behavior of each channel also depending on the spectral characteristics of the sample to be measured. While the intensity measures of reflected light on the four wavelengths are not comparable even in the same acquisition, comparability remains unchanged with regard to the reflectance measures obtained in post-processing.

### 3.2. Calibration

The calibration procedure was designed on the basis of the considerations about the characteristic curves of the system, as a function of the Gain and Stimulus parameters.

Regarding the amplification Gain parameter, the above considerations (see Section 2.4) show that G=1 is the optimal value, independent of the sample and the application. The Gain parameter was therefore determined *una tantum* and preset at this fixed value.

On the other hand, for the Stimulus parameter, a dedicated algorithm implemented in the microcontroller automatically searches and sets the optimum for each channel, every time the instrument is switched on.

**Figure 4:**
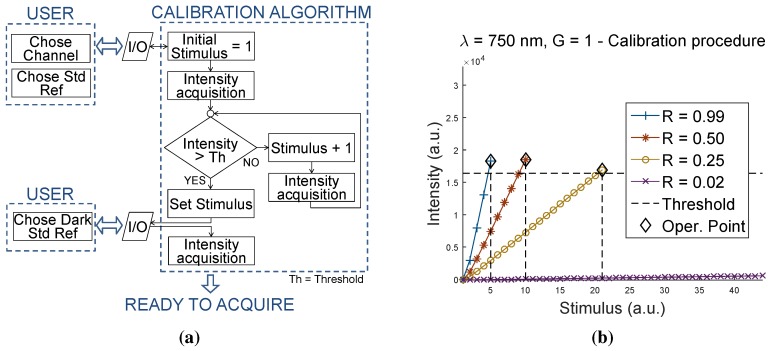
(**a**) Block diagram for the calibration procedure along a generic channel; (**b**) results of calibration on channel λ=750 nm, using standard references R=0.99,0.50,0.25,0.02.

According to this algorithm, an initial acquisition on a standard reference chosen by the user must be conducted. As schematically shown by the diagram in Figure 4a, the lowest level of Stimulus that produces a detected light intensity above an appropriate threshold is determined through the successive incremental Stimulus signal. As a detected intensity threshold, the half value of the full available scale (32768/2 a.u.=16384 a.u.) was chosen. If calibrating with an appropriate standard reference, this threshold value avoids the risk of saturation during a working session, while ensuring a sufficient resolution to capture the dynamics of all expected samples.

The value of Stimulus, which is defined in the calibration for each channel, is then used to power the corresponding LED during the whole working session, until the instrument is switched off. In this way, the user has only an indirect effect on setting the optimal Stimulus for his/her specific application (*i.e.*, with the choice of the reference used in the calibration). Once the Stimulus level is determined, an acquisition of a dark reference (R=0.02) concludes the calibration procedure.

Figure 4b presents the result obtained with this procedure on channel λ=750 nm for a calibration with the standard references R=0.99,0.50,0.25,0.02. The sequential increments of the detected light intensity produced by the increasing Stimulus signal are shown. The algorithm stops as soon as the threshold value is exceeded, and the last value of Stimulus is thus set to control the corresponding LED during all working sessions.

The plots of Figure 4b also highlight how the slope of the line changes with the standard reference used in the calibration, which results in different values determined by the algorithm for the Stimulus parameter. As an illustrative example, the attempt to calibrate with the dark standard reference (R=0.02) is also shown. As expected, this resulted in the impossibility to reach the threshold value by the measured intensity.

These results show how the only thing the operator has to pay attention to is the choice of the standard reference when calibrating. According to this procedure, the reference should provide a spectral reflectance as similar as possible to the ones of the samples to be measured, ensuring a proper positioning of the operation point along the characteristic curve for the specific application.

The same calibration procedure has to be repeated for each channel, *i.e.*, for each LED, by using appropriate standard references with respect to the spectral characteristic of the samples, in order to optimize the acquisition along every channel.

### 3.3. Response and Validation Tests on Standard Solutions

**Figure 5:**
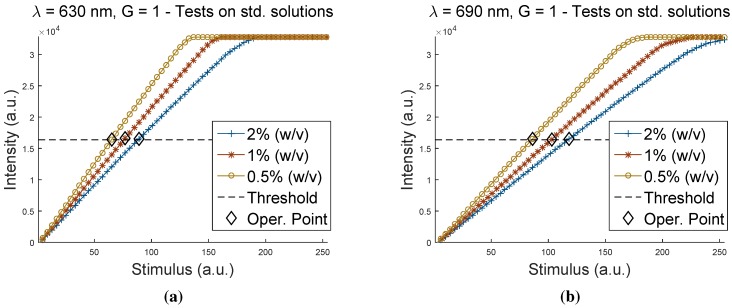
Output Intensity *vs.* Stimulus with the Gain parameter G=1 for acquisitions on aqueous solutions of blue dye at different concentrations: (**a**) channel at λ=630 nm; and (**b**) channel at λ=690 nm.

Figure 5 presents the results coming from the tests on the aqueous solutions of blue dye at concentrations of 0.5% (w/v), 1% (w/v) and 2% (w/v), respectively. The curves of the output intensity measured as a function of Stimulus variable for the three samples are shown together in the same plot (the Gain parameter is now set at the fixed value G=1, previously determined). For the sake of clarity, only the results for the two visible channels are presented (λ=630 nm at the left and λ=690 nm at the right), since these are the most critical spectral bands for dark samples and the ones that we wanted to investigate. Data referring to other channels are accessible as supplementary material, with the measurement files in Appendix E, Appendix F and Appendix G.

As expected from the previous results, the curves show an excellent linearity and have a slope that decreases with the increasing concentration of the dye. The slope is therefore consistent with the degree of the darkness of the sample. This proves the capability of the device to discriminate between dark samples, which are apparently similar in a visible sense, by detecting the correspondent differences in spectral reflectance. This is the typical situation of dark fruits, like berries of red grape or blueberries.

On the basis of this and previous considerations, the solutions at different concentrations of dye could be used as handmade and cost-effective references to calibrate the instrument for measurements on dark samples. Regarding this possibility, the figure shows the horizontal threshold line and the operation point (see the diamond markers), which should be identified by calibrating the instrument with the solutions. As shown, the use of these kinds of handmade references makes it possible to set high values for the Stimulus parameter, otherwise unreachable, particularly suitable for measuring dark fruits. A wider and more detailed range of references could be realized according to a simple protocol and then replicated when needed, depending on the specific application.

**Table 3:** Validation measurements on standard water solutions of blue dye at concentrations of 0.5% (w/v), 1% (w/v) and 2% (w/v), respectively. Measurements on five repetitions in the form of AverageIntensity±StandardDeviation are presented. Abbreviations used in the table: Calib. = Calibration; Std. = Standard.

Channel	Reference Used in Calibration	Calib. Setpoint	Measurements (Intensity[a.u.]) on Std. Solutions at		
λ[nm]		Stimulus[a.u.]	0.5% (w/v)	1% (w/v)	2% (w/v)
630	Std. solution, blue dye 1% (w/v)	77	19,433±653	16,577±369	14,273±447
690	Std. solution, blue dye 1% (w/v)	103	19,636±719	16,447±387	14,196±519
750	Std. reference, R=0.25	23	11,741±276	12,996±312	13,725±389
850	Std. reference, R=0.50	14	7962±231	8945±289	10,966±300

The results coming from the final validation tests of the system in its operative configuration are shown in Table 3. To take into account measuring dark fruits as a possible application, the 1% (w/v) standard solution was used as a reference for calibrating both visible channels (λ=630,690 nm). Conversely, in the near-infrared spectral bands, the calibration was conducted with the standard reference R=0.25 for the channel λ=750 nm, and the standard reference R=0.50 for the channel λ=850 nm, respectively. As desired, the calibration procedure consistently returned a different value for the Stimulus parameter, specific for every channel.

For the three measured samples, *i.e.*, the three water standard solutions, the average measured intensities and the standard deviations on five different acquisitions are reported. The data again are consistent with the degree of the darkness of the acquired standard solution, confirming the capabilities of the device for discriminating very low levels of reflectance. These tests also prove the measurement repeatability of the device system: indeed, the standard deviations are proportional to the measured intensity and in general limited to 2%–4% of the measured value.

## 4. Conclusions

In this work, a prototype of a simplified optical VIS/NIR device based on LED technology was designed and tested. Laboratory tests were conducted for the study of the characteristic curve of the instrument. In particular, the system response as a function of the two setting parameters was investigated: the Gain amplification factor and the Stimulus level for the drive signal of the LEDs. In both cases, a highly linear response was obtained.

The optimal setting value for the amplification parameter has been determined as Gain=1. This is accompanied by a semi-automatic calibration procedure implemented to allow the user to define an optimal Stimulus level for each LED driving signal independently. The system can therefore emit a custom four-wavelength spectrum, optimized for the specific application. In this way and thanks to the modularity given by the LEDs/filters units, the same device could be adapted for measuring different products and for a wide range of applications.

The sensitivity of the device has highlighted the need for additional references. A simple and economic solution could be the definition of a protocol for the preparation of custom references. In this work, water solutions of standard food dyes at different concentrations were proposed.

Final validation tests on these kind of solutions prove the measurement repeatability of the device with a standard deviation limited to 2%–4% and confirmed its capabilities of discriminating low levels of reflectance. This could be particularly useful when measuring dark fruits, like berries of red grape or blueberries.

The integration of a simple processing algorithm in the microcontroller software would allow one to visualize real-time values of reflectance. An evolution and an engineering phase is desirable in order to obtain a handheld, user-friendly and inexpensive device for farmers’ utilization in the field.

## A. Acquisition File on Standard Reference R=0.99

Acquisition .txt file for a measurement on the standard reference R=0.99, when using the instrument in the explorative configuration mode for the evaluation of its characteristic curve.

## B. Acquisition File on Standard Reference R=0.50

Acquisition .txt file for a measurement on the standard reference R=0.50, when using the instrument in the explorative configuration mode for the evaluation of its characteristic curve.

## C. Acquisition File on Standard Reference R=0.25

Acquisition .txt file for a measurement on the standard reference R=0.25, when using the instrument in the explorative configuration mode for the evaluation of its characteristic curve.

## D. Acquisition File on Standard Reference R=0.02

Acquisition .txt file for a measurement on the standard reference R=0.02, when using the instrument in the explorative configuration mode for the evaluation of its characteristic curve.

## E. Acquisition File on the Standard Solution at 0.5%(w/v)

Acquisition .txt file for a measurement on the aqueous solution of blue dye at a concentration of 2% (w/v), when using the instrument in the explorative configuration mode for the evaluation of its characteristic curve at Gain=1.

## F. Acquisition File on the Standard Solution at 0.5%(w/v)

Acquisition .txt file for a measurement on the aqueous solution of blue dye at a concentration of 1% (w/v), when using the instrument in the explorative configuration mode for the evaluation of its characteristic curve at Gain=1.

## G. Acquisition File on the Standard Solution at 0.5%(w/v)

Acquisition .txt file for a measurement on the aqueous solution of blue dye at a concentration of 0.5% (w/v), when using the instrument in the explorative configuration mode for the evaluation of its characteristic curve at Gain=1.
